# Hypothermia and temperature modulation for intracerebral hemorrhage (ICH): pathophysiology and translational applications

**DOI:** 10.3389/fnins.2024.1289705

**Published:** 2024-02-19

**Authors:** Angel J. Cadena, Fred Rincon

**Affiliations:** ^1^Department of Neurology, Columbia University, New York, NY, United States; ^2^Department of Neurology, Division of Neurocritical Care, Cooper University, Camden, NJ, United States

**Keywords:** intracerebral hemorrhage, ICH, hypothermia, normothermia, cryotherapy, secondary brain injury, hypothermia in ICH, inflammation

## Abstract

**Background:**

Intracerebral hemorrhage (ICH) still poses a substantial challenge in clinical medicine because of the high morbidity and mortality rate that characterizes it. This review article expands into the complex pathophysiological processes underlying primary and secondary neuronal death following ICH. It explores the potential of therapeutic hypothermia as an intervention to mitigate these devastating effects.

**Methods:**

A comprehensive literature review to gather relevant studies published between 2000 and 2023.

**Discussion:**

Primary brain injury results from mechanical damage caused by the hematoma, leading to increased intracranial pressure and subsequent structural disruption. Secondary brain injury encompasses a cascade of events, including inflammation, oxidative stress, blood-brain barrier breakdown, cytotoxicity, and neuronal death. Initial surgical trials failed to demonstrate significant benefits, prompting a shift toward molecular mechanisms driving secondary brain injury as potential therapeutic targets. With promising preclinical outcomes, hypothermia has garnered attention, but clinical trials have yet to establish its definitive effectiveness. Localized hypothermia strategies are gaining interest due to their potential to minimize systemic complications and improve outcomes. Ongoing and forthcoming clinical trials seek to clarify the role of hypothermia in ICH management.

**Conclusion:**

Therapeutic hypothermia offers a potential avenue for intervention by targeting the secondary injury mechanisms. The ongoing pursuit of optimized cooling protocols, localized cooling strategies, and rigorous clinical trials is crucial to unlocking the potential of hypothermia as a therapeutic tool for managing ICH and improving patient outcomes.

## 1 Introduction

Intracerebral hemorrhage (ICH) accounts for 10–20% of all strokes (Rincon and Mayer, 2012, 2013; Cui et al., 2015), representing a significant challenge characterized by increased morbidity and mortality. While constituting a relatively small portion of all strokes, ICH disproportionately impacts health outcomes as approximately one-third of patients with ICH do not survive the initial event (Han et al., 2017; Wilkinson et al., 2018; Broderick et al., 2021; Baker et al., 2022a). Only 20% of the survivors regain functional independence at 6 months, contributing to a substantial burden on the patients, caregivers, and healthcare systems (Mayer and Rincon, 2005; Broderick et al., 2021).

Complex pathophysiologic processes after ICH can be divided into primary and secondary events leading to brain injury that contribute to its devastating effects. Primary brain injury arises from direct mechanical damage caused by the hematoma, disrupting surrounding structures and elevated intracranial pressure (Baker et al., 2022b). Secondary brain injury comprises a cascade of events, including activation of the inflammatory cascade, generation of free radicals, iron release, oxidative stress, blood-brain barrier breakdown, cytotoxicity, peri-hematomal edema, and neuronal death (Mayer and Rincon, 2005; Aronowski and Zhao, 2011; Zhou et al., 2014; Cui et al., 2015; Xiong and Yang, 2015; Baker et al., 2022b).

As hematoma volume and expansion are critical factors in determining outcomes, initial research has focused on early hematoma evacuation (Zhou et al., 2014). However, while addressing hematoma evacuation, surgical trials (Mendelow et al., 2005, 2013; Hanley et al., 2019) did not prove significant results, prompting a shift in scientific interest toward understanding the intricate molecular mechanisms driving secondary brain injury as potential targets for therapeutic intervention such as therapeutic hypothermia.

As an innovative therapeutic option, hypothermia has garnered attention for its potential to disrupt the pathological processes underlying secondary brain injury. Promising outcomes have been observed in preclinical studies in which hypothermia was found to mitigate tissue damage and improve outcomes (Melmed and Lyden, 2017; Baker et al., 2022a). Nevertheless, despite these promising preclinical findings, clinical trials have yet to yield conclusive evidence of hypothermia’s effectiveness in treating ICH-related complications (Baker et al., 2022a).

Given the persistently grim outcomes associated with ICH, there is an urgent need to explore novel therapeutic approaches that can mitigate secondary neuronal damage and cerebral edema. This comprehensive review aims to consolidate existing knowledge regarding therapeutic temperature modulation’s role in managing ICH, specifically hypothermia. This review seeks to advance therapeutic strategies for this highly challenging condition by critically assessing the available literature.

## 2 Materials and methods

We comprehensively reviewed the literature of relevant studies published between 2000 and 2023, synthesizing information on hypothermia in the context of intracerebral hemorrhage (ICH). Search strategies: The authors searched the literature independently, utilizing several prominent databases, including PubMed, Medline, Cochrane Database of Systematic Reviews, and Cochrane Central Register of Controlled Trials (CENTRAL). We employed a systematic search strategy incorporating Medical Subject Headings (MeSH) keywords to identify studies pertinent to our research. The following MeSH keywords were used: “Hypothermia and Intracerebral hemorrhage,” “Cryotherapy and ICH,” “Management of ICH,” “Intracerebral hemorrhage and brain injury,” “Secondary brain injury and ICH,” and “Surgical management and ICH.” Eligibility criteria: To meet inclusion criteria, articles had to be published within the specified timeframe, with a focus on a subject population with spontaneous intracerebral hemorrhagic stroke (specifically intraparenchymal hemorrhage), and that discussed the use of surgical evacuation of the hematoma and hypothermia as therapeutic approaches. Other therapeutic approaches were considered if the article also provided an explanation of the pathophysiology of ICH, but the primary focus was therapeutic hypothermia. Exclusion criteria encompassed traumatic ICH, as it falls under traumatic brain injury (TBI) with distinct pathophysiology, and our review specifically targets primary ICH (related to hypertension and amyloid angiopathy). No language restrictions were applied during the search process. In cases of disagreement among the authors regarding the inclusion of an article, consensus was reached through discussion. Study selection: The database search initially identified approximately 14,000 articles. After removing duplicates, those inaccessible or with only available abstracts, and after applying the inclusion criteria, 75 articles remained. Due to their significance, three more articles were added later, resulting in a final count of 78 articles used for this study.

## 3 Pathophysiology

### 3.1 Primary brain injury

Primary brain injury following ICH includes the deleterious consequences of the mechanical pressure exerted by extravasated blood on adjacent cerebral structures (Baker et al., 2022a). The ensuing sequelae mainly comprise hematoma and edema expansion, exacerbating neurological deterioration and functional impairment (Han et al., 2017). The inciting factors behind primary injury manifestations, particularly early hematoma expansion, entail the breakdown of the blood-brain barrier integrity, ischemic infarctions, and localized coagulopathy (Mayer and Rincon, 2005). Concomitantly, the increase in intracranial pressure compromises cerebral perfusion, engendering shear forces that lead to micro-bleed phenomena, fostering hematoma enlargement and accentuating the vulnerability to cerebral herniation (Mayer and Rincon, 2005; Keep et al., 2012).

The rationale that early hematoma evacuation could mitigate neurological deterioration and improve functional outcomes led to considerable research to elucidate the efficacy of early surgical intervention. However, no randomized clinical trial (Broderick et al., 2021) addressing early surgical evacuation has shown any significant benefit. In 2005 and 2013, The Surgical Treatment for Intracerebral Hemorrhage (STICH-I) (Mendelow et al., 2005) and STICH-II (Mendelow et al., 2013) trials compared early survival evacuation via craniotomy versus medical management without finding any significant benefits. Later, the results of the Minimally Invasive Surgery with Thrombolysis in ICH Evacuation (MISTIE-III) (Hanley et al., 2019) trial comparing minimally invasive surgery with thrombolysis in ICH evacuation was published in 2019 and showed no difference in functional outcomes. However, in MISTIE-III, a subgroup of patients who demonstrated benefit from hematoma evacuation with this technique were those who achieved a residual hematoma of less than 15 ml.

### 3.2 Secondary brain injury

**FIGURE 1:**
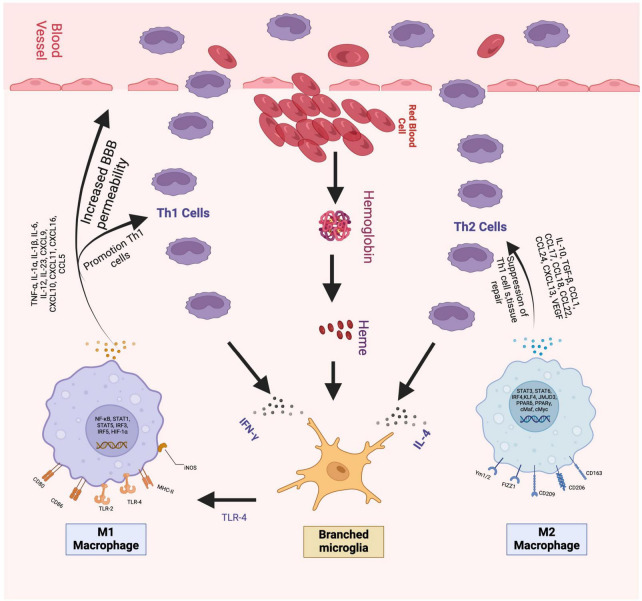
The figure shows the beginning of the cellular inflammation process. On the left is the pro-inflammatory part, which begins with the activation of M1 cells followed by the release of various cytokines, especially IL-1, TNF-alpha, and IFN gamma, which exacerbate cellular injury and damage to the blood-brain barrier. To the right of the Figure, you can see the anti-inflammatory portion represented by M2 cells. M2 cells release IL-4, IL-10, and TGF-beta, among other Citokynes shown in the picture, which decrease the expression of Th1 cells and contribute to cell repair. Figures were made by the authors.

There are intricate and multifaceted molecular and cellular processes that occur immediately after the formation of the hematoma, including the activation of the inflammatory cascade, the release of cytokines, and the induction of oxidative stress, all of which characterize secondary brain injury (see Figure 1) and contribute to neuronal degeneration (Baker et al., 2022a). Essential components like coagulation factors, iron, thrombin, and red blood cells can initiate neuronal damage, as evidenced by peri-hematomal edema (Aronowski and Zhao, 2011; Babu et al., 2012; Keep et al., 2012; Baker et al., 2022b).

#### 3.2.1 Oxidative stress

Upon hematoma formation, lysed red blood cells release hemoglobin and its degradation products (Wagner et al., 2003; Hua et al., 2007; Kearns et al., 2021). The free heme molecule precipitates within brain tissue, subsequently undergoing oxidation to hemin, accompanied by the release of excess iron and bilirubin, depletion of glutathione, and generation of free radicals (Robinson et al., 2009; Babu et al., 2012). This oxidative stress correlates with brain tissue atrophy and aggravated cerebral edema (Nakamura et al., 2004; Aronowski and Zhao, 2011; Babu et al., 2012). A meta-analysis of preclinical studies demonstrated the potential of reducing brain edema by administering an iron chelator, particularly within the critical window of 2–4 h (Cui et al., 2015). Therapeutic hypothermia attenuates oxidative stress and reduces the generation of free radicals by inhibiting superoxide and lipid peroxidation, primarily if mild hypothermia (32–35 C) is applied (Gao et al., 2014; Liu and Zhou, 2023).

#### 3.2.2 Thrombin, complement cascade, and inflammatory cytokines

Thrombin functions as a protease, and its level increases within the initial hours following hematoma formation (Hua et al., 2007; Aronowski and Zhao, 2011; Askenase and Sansing, 2016). It plays a role in cleaving C3 and C4, subsequently activating the complement cascade, which triggers the release of chemotactic factors for leukocytes (Ducruet et al., 2009; Babu et al., 2012). Crucially, thrombin binds to protease-activated receptors (PARs), whose ICH elevation increases blood-brain barrier permeability and excitotoxicity (Donovan et al., 1997; Fujimoto et al., 2007; Baker et al., 2022b). Furthermore, thrombin has been identified as a catalyst for elevating intracellular calcium levels in microglia, prompting the release of Nitric Oxide (NO), TNF-alpha, IL-2, and IL-6, all of these are pro-inflammatory cytokines (see Figure 1) which contribute to the exacerbation of cerebral edema (Holmin and Mathiesen, 2000; Wu et al., 2008; Zhou et al., 2014). Preclinical studies using systemic or focal hypothermia showed decreased thrombin-induced cerebral edema. However, other studies did not find that hypothermia reduced thrombin-induced neurotoxicity (Kawai et al., 2001; Gao et al., 2014; Wowk et al., 2014).

#### 3.2.3 Cell activation, inflammation, and neuronal death

**FIGURE 2:**
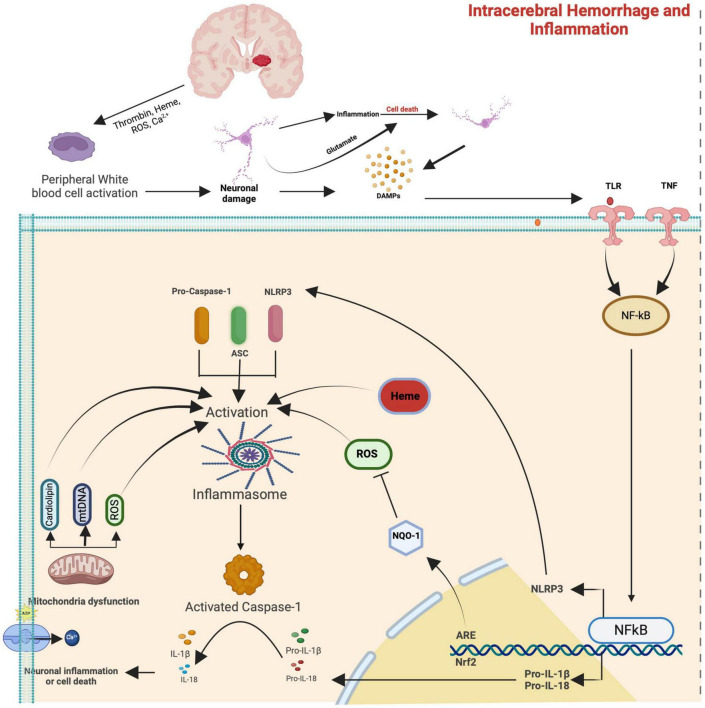
Simplified signaling and the inflammasome pathways that contribute to secondary brain damage after spontaneous cerebral hemorrhage. The upper part of the image is a simplification from Figure 1 (Please refer to), which shows that after ICH degradation products from the blood (hemoglobin, heme, and thrombin) and ROS can lead to peripheral activation, but also direct neuronal damage and subsequent release of damage-associated molecular pattern molecules (DAMPs) that interact with Toll-like receptor (TLR) and tumor necrosis factor receptor (TNFR) starting the inflammasome cascade with the activation of nuclear factor kappa B (NF-κB). The NF-κB then goes to the nucleus and binds to the DNA, promoting the expression of NLRP3, pro-IL-1β, and pro-IL-18 proteins. Then, mitochondrial membrane damage with the later release of ROS, mitochondrial DNA, and cardiolipin exacerbates cellular injury, leading to cell death.

In response to the degradation products of the hematoma, microglia, the resident phagocytes of the central nervous system (CNS), undergo rapid activation within minutes (Aronowski and Hall, 2005; Wang, 2010; Rolland et al., 2013). These activated microglia subsequently release a spectrum of pro-inflammatory cytokines (IL-1β, IL-6, IL-18, and tumor necrosis factor (TNF), chemokines, and free radicals, facilitating the infiltration of other inflammatory cells, including leukocytes, macrophages, and T cells (Aronowski and Hall, 2005; Wagner, 2007), thereby intensifying secondary brain injury (Aronowski and Hall, 2005). The nuclear transcription factor kappa-B (NF-κB) is instrumental in mediating these inflammatory signals (see Figure 2; Aronowski and Zhao, 2011).

Several mechanisms are proposed for microglia activation, encompassing Toll-like Receptor (TLR) signaling pathways, CD36-mediated activation, thrombin signaling, and complement activation (Moller et al., 2000; Aronowski and Hall, 2005). Toll-like receptors (TLRs) are transmembrane receptors that recognize damage-associated molecular patterns (DAMPs), instigating inflammation in response to injury (Kong and Le, 2011). Notably, increased TLR2 and TLR4 (see Figure 2) expression in monocytes has been linked to unfavorable outcomes in ICH (Rodriguez-Yanez et al., 2012). During cell demise, danger-associated molecular patterns (DAMPs) are released, initiating signals that prompt leukocyte infiltration, furthering secondary neuronal death (Zhou et al., 2014).

Within 24 h, neutrophils emerge as the earliest leukocytes within the hematoma (Gong et al., 2004), and their elevated levels are a risk factor for premature neurological deterioration after ICH (Leira et al., 2004). The absence of CD8 and CD4 cells in stroke models has shown improved outcomes, underscoring their contribution to the inflammatory processes (Yilmaz et al., 2006). Therapeutic hypothermia decreases the inflammatory response by inhibiting pro-inflammatory markers, potentially contributing to reduced perihematoma edema (Lee et al., 2016; Baker et al., 2022b).

The culmination of the inflammatory cascade leads to secondary neuronal death, occurring via either apoptosis or necrosis (Elmore, 2007). Following cell demise, whether through apoptosis or necrosis, the release of DAMPs signals an inflammatory feedback loop that further cyclically exacerbates secondary neuronal loss (Kono, 2008). Mild hypothermia inhibits apoptosis by down-regulating the pro-apoptotic BAX gene expression and reducing the production of caspase-8 through decreased matrix metalloproteinase (MMP) expression (Deng et al., 2003; Lee et al., 2005, 2016). Additionally, there is an observed increase in the expression of the antiapoptotic protein BCL-2, which reduces neuronal cell death.

In some clinical studies, the impact of hypothermia on inflammation has not been definitively established. Two treatment strategies were compared in a controlled clinical trial (Broessner et al., 2010) involving 102 patients with severe cerebrovascular disease [including spontaneous subarachnoid hemorrhage (SAH), spontaneous ICH, or complicated cerebral infarction (AIS)]. Patients were either subjected to prophylactic endovascular long-term normothermia or conventional stepwise fever management involving anti-inflammatory drugs and surface cooling. The study’s findings indicated that the group treated with endovascular normothermia significantly increased pro-inflammatory markers, while these markers decreased in the group managed conventionally.

In another clinical trial (Fischer et al., 2012), the levels of endothelial markers, angiopoietin-1 and -2, were measured in patients with spontaneous SAH or ICH, randomized to either normothermia through an endovascular device or fever control using antipyretic medication and surface cooling. The study did not find any significant difference in the levels of these markers on days 1, 4, and 7. Notably, when the intervention group received non-steroidal anti-inflammatory drugs (NSAIDs), it led to a significant decrease in angiopoietin-2 levels on day 7, which is known to destabilize the vascular endothelium.

#### 3.2.4 Fever

Fever is prevalent in approximately 90% of patients with ICH (Schwarz et al., 2000), particularly when associated with intraventricular hemorrhage (IVH) (Commichau et al., 2003; Mayer and Rincon, 2005). It increases brain metabolism and accelerates secondary neuronal death, promoting hematoma expansion and poorer outcomes, even with minor temperature elevations (Mayer and Rincon, 2005; Rincon et al., 2013). Implementing temperature control measures may lead to better outcomes following ICH (Rincon et al., 2013); however, clinical trials have not shown conclusive results on long-term outcomes (Baker et al., 2022a). The recently closed Impact of Fever Prevention in Brain-Injured Patients (INTREPID) study failed to demonstrate a significant effect of prophylactic temperature modulation to reduce fever in stroke patients, including ICH (Greer et al., 2021).

#### 3.2.5 Cerebral autoregulation

Cerebral autoregulation functions as a protective mechanism that ensures a consistent cerebral blood flow (CBF) throughout different levels of blood pressure, preventing both hypoperfusion and hyperperfusion (Oeinck et al., 2013; Liu and Zhou, 2023). Myogenic, neurogenic, and endothelial factors regulate CBF (Rivera-Lara et al., 2017). However, these mechanisms are effective only for a limited duration and are easily overwhelmed following acute brain injury (Liu and Zhou, 2023). In ICH, impaired cerebral autoregulation is common and often leads to unfavorable outcomes (Ma et al., 2016; Liu and Zhou, 2023). Studies indicate cerebral autoregulation is particularly impaired in the initial days after ICH, but it experiences incomplete recovery after 30 days (Nakagawa et al., 2011; Ma et al., 2016, 2017). As previously mentioned, hypothermia plays a vital role in reducing metabolic demand, oxidative stress, and inflammation and maintaining the integrity of the blood-brain barrier, all of which may significantly influence cerebral autoregulation (Liu and Zhou, 2023). Experimental studies have demonstrated regulated cerebral perfusion during exposure to hypothermia and dysregulation during the transition from hypothermia to normothermia, indicating potential clinical implications for temperature modulation in ICH (Lavinio et al., 2007).

## 4 Pre-clinical data

In pre-clinical research, various methodologies are employed to induce ICH, an autologous blood injection or collagenase administration, which are the most widely utilized approaches (Xue and Del Bigio, 2000; Askenase and Sansing, 2016; Baker et al., 2022a).

In the collagenase model, collagenase injection into the striatum dissolves the extracellular matrix surrounding the blood vessel endothelia, leading to a spontaneous ICH in the basal ganglia (Xue and Del Bigio, 2000; James et al., 2008; Barratt et al., 2014). This model offers advantages in reproducibility and control of the hematoma size. However, a notable drawback lies in the potential overstimulation of the immune response, attributed to the bacterial origin of collagenase, leading to the activation of downstream events, including exacerbated perihematomal cerebral edema (Xue and Del Bigio, 2000; James et al., 2008; Barratt et al., 2014; Askenase and Sansing, 2016). Conversely, the autologous blood ICH model involves blood injection, typically into the striatum. One key advantage of this method is its precise control over the hematoma size. Nevertheless, it does not recreate the endothelial breakdown and the associated inflammatory responses nor facilitates the exploration of re-bleeding scenarios (James et al., 2008; Askenase and Sansing, 2016).

Melmed and colleagues conducted a comprehensive meta-analysis (Melmed and Lyden, 2017) encompassing 18 preclinical studies centered on hypothermia as a therapeutic approach for ICH utilizing mainly autologous blood and collagenase models. These studies employed a target temperature range of 32°C to 35°C and encompassed diverse cooling techniques, such as whole-body and selective cooling via implantable intracranial devices. Many studies used local cooling, representing 42.8% (10 studies). Notably, this analysis unveiled compelling evidence that therapeutic hypothermia exerts beneficial effects across multiple fronts, including mitigation of cerebral edema, attenuation of blood-brain barrier breakdown, and improved behavioral outcomes. Remarkably, these positive outcomes were held despite the inherent study heterogeneity and the variations in cooling methodologies applied. Subsequently, Baker and colleagues validated these findings through an additional systematic review and meta-analysis (Baker et al., 2022a), lending further support to the potential effect of hypothermia as a therapeutic avenue for managing the impact of the pathophysiological effects related to ICH.

## 5 Local and systemic therapeutic hypothermia

Therapeutic hypothermia (TH) induces lower body temperatures than the standard parameter (37–38°C). It has been traditionally classified according to the degree of cooling in mild (32-35°C), moderate (28–32°C), severe (20–28°C), and profound hypothermia (≤ 20°C) (Liu and Zhou, 2023). TH emerges as a versatile intervention, illustrating its potential to mitigate neuronal injury across multiple acute and devastating neurological conditions, including ischemic stroke (Kollmar and Schwab, 2009; Dumitrascu et al., 2016) and ICH (Melmed and Lyden, 2017; Baker et al., 2022a).

The neuroprotective effect of TH in intracerebral hemorrhage is grounded in its ability to impact multiple molecular and cellular targets, such as a reduction of the metabolic demand, a decrease of inflammatory markers (TNF-α, IL-1β, IL-6, IL-18) and modulation of the immune response, upregulation of anti-apoptotic proteins such as BCL-2, and attenuation of free radical generation (Han et al., 2017; Melmed and Lyden, 2017; Baker et al., 2022b; Liu and Zhou, 2023).

In systemic hypothermia, the target is to reduce the body-core temperature, involving all the organs through several methods, such as surface or endovascular cooling (Baker et al., 2022b). Both methods appear to have no apparent difference in efficacy or targeting temperature (Kim et al., 2018).

However, systemic hypothermia entails a set of potential risks, spanning from shivering and increased infection rates to coagulopathy and arrhythmias. Notably, an impending concern in animal models is the conceivable risk of hematoma expansion, attributed to its potential influence on the coagulation cascade (Aronowski and Zhao, 2011; Baker et al., 2022a). Due to this limitation, the scientific community has gained interest in local and regional colling systems that would induce local hypothermia (Zhao et al., 2018; Baker et al., 2022a,b) (e.g., In the brain), but maintain core normothermia (Baker et al., 2022b Consensus) via avenues such as intranasal cooling, intra-carotid cooling, and the utilization of specialized helmets, all of which have demonstrated efficacy in animal models (Melmed and Lyden, 2017; Baker et al., 2022a). This strategic shift toward localization strives to maximize the benefits of hypothermia and mitigate the potential drawbacks associated with a systemic approach.

## 6 Clinical data

### 6.1 Observational studies

Schwarz and colleagues conducted a study to assess the prognostic significance of fever during presentation and within the subsequent 72 h in patients with spontaneous supratentorial intracerebral hemorrhage (ICH). Their findings indicated that the duration of fever was an independent prognostic risk factor associated with poor outcomes (Schwarz et al., 2000).

In a retrospective cohort study (Rincon et al., 2013), Rincon and colleagues found that fever was a common occurrence following ICH, and it held its significance even when adjusting for infection and baseline hematoma volume. Their findings revealed that fever was independently associated with hematoma expansion and poor functional outcomes. In a case-control study, the authors explored the impact of therapeutic hypothermia, focusing on the timing of its initiation. Interestingly, the study demonstrated that initiating hypothermia early was linked to reduced perihematomal cerebral edema. Notably, the duration of hypothermia (short, 4–8 days, or long, 9–15 days) and the rewarming time did not significantly differ in their effects on the peri-hematomal cerebral edema evolution (Volbers et al., 2016). These findings provide valuable insights into the potential benefits of ultra-early targeted temperature management, particularly considering the heightened inflammatory state immediately following hematoma formation.

### 6.2 Clinical trials

Concrete evidence supporting the effectiveness of medical interventions to enhance outcomes in cases of intracerebral hemorrhage still needs to be discovered. Multiple surgical trials centered around hematoma evacuation have not provided substantial proof of significant benefits. Consequently, the spotlight has turned toward therapeutic hypothermia as a potential avenue for mitigating secondary brain injury. This shift is rooted in the potential benefits consistently demonstrated in preclinical studies. However, translating these promising findings into clinical trials has encountered obstacles, with the anticipated benefits yet to be definitively established.

Historically controlled studies such as the one done by Staykov et al. (2013) have demonstrated the potential of cooling to mitigate peri-hematomal cerebral edema in patients with intracerebral hemorrhage (ICH). However, these studies have also underscored the emergence of increased adverse events, including a heightened risk of infection, notably pneumonia, shivering, and bradyarrhythmia. Regrettably, these benefits in edema reduction have not translated into tangible improvements in functional outcomes. Conversely, two distinct investigations have yielded more promising results: one delving into localized hypothermia (Su et al., 2015) and another assessing the impact of cooling and hematoma evacuation (Zhao et al., 2018), showcasing improvements in functional outcomes.

The Targeted temperature management after intracerebral hemorrhage (TTM-ICH) trial (Rincon et al., 2014) represented the first randomized clinical trial in the United States dedicated to exploring the effects of therapeutic hypothermia in the context of ICH. This pivotal trial assessed the viability of an ultra-early protocol for temperature modulation and its impact on clinical outcomes. Enrolled patients were randomized within an 18-h window from symptom onset and allocated to either a moderate hypothermia group (32–34°C) for 72 h, followed by controlled rewarming, or a normothermia group (36–37°C), facilitated via endovascular or surface cooling. The results of this study are forthcoming. Similarly, the Cooling in intracerebral hemorrhage (CINCH) trial (Kollmar et al., 2012) emerges as another prospective, multicenter randomized endeavor, spanning across Germany and Austria, with the primary objective of assessing the influence of therapeutic hypothermia on survival in cases of ICH.

### 6.3 Systematic review and meta-analysis

Baker et al. (2022a) performed a systematic review and meta-analysis to scrutinize the impact of therapeutic hypothermia after ICH, encompassing a comprehensive array of twenty-one preclinical and five clinical studies. As delineated earlier, the preclinical studies showed significant benefits in behavioral outcomes, cerebral edema, and blood-brain barrier integrity, all achieved without concomitant hematoma expansion. The clinical studies, however, were characterized by pronounced heterogeneity, precluding the possibility of a meta-analysis. This divergence among studies emanated from disparities in cooling techniques, initiation protocols, duration of intervention, target temperature ranges, and the scope of outcome measurements. Interestingly, Su et al. (2015) embarked on an assessment of localized hypothermia, employing a head wrap strategy, which showed significant improvement in functional outcomes. Equally compelling, another study (Campbell et al., 2018) explored the synergistic effect of systemic hypothermia through surface cooling, coupled with ICH evacuation, leading to improved NIH Stroke Scales scores and noteworthy reductions in inflammatory biomarkers such as TNF-alpha and NF-kB; however, the follow-up was limited to 7 days.

## 7 Discussion

Intracerebral hemorrhage (ICH) remains a significant clinical challenge due to its high morbidity, mortality, and disproportionately negative impact on patient outcomes (Sacco et al., 2009; Rincon and Mayer, 2012, 2013; Cui et al., 2015). This public health issue highlights the need to explore novel therapeutic strategies to mitigate the devastating effects of the complex pathophysiologic mechanisms behind both primary and secondary neuronal injury (Broderick et al., 2021; Baker et al., 2022a,b). To this end, we reviewed the role of cryotherapy, specifically therapeutic hypothermia (both systemic and local), for ICH, considering its molecular and translational applications.

The Initial enthusiasm for hematoma evacuation arose from the potential to mitigate its mechanical effects on surrounding structures. However, despite initial research efforts, early surgical evacuation has yet to demonstrate significant benefits in improving outcomes, as evidenced by trials such as STICH-I, (Mendelow et al., 2005), STICH-II (Mendelow et al., 2013), and MISTIE-III (Hanley et al., 2019). The failure of these trials to achieve their primary endpoints can be attributed in part to the substantial crossover between treatment arms and heterogeneity in protocol application, precisely efficiency in clot removal, and residual hematoma volume. Additionally, the timing of surgical intervention from symptom onset played a crucial role, with an average delay of 30 h, 27 h, and 58 h for STICH-I, STICH-II, and MISTIE-III, respectively (Kellner et al., 2021). This raises important questions about the potential significance of ultra-early evacuation as a critical factor in future surgical trials.

The neutral outcomes observed in these surgical trials have prompted a paradigm shift toward a more multi-modal or comprehensive approach utilizing a molecular matrix, targeting intricate mechanisms underpinning secondary neuronal injury. This complex cascade of events encompasses inflammation, oxidative stress, blood-brain barrier breakdown, cytotoxicity, and neuronal death (Aronowski and Zhao, 2011; Babu et al., 2012; Keep et al., 2012; Baker et al., 2022a). Additionally, the presence of pro-inflammatory cells, particularly the early elevation of neutrophils, has been identified as a factor leading to early neurological deterioration (Leira et al., 2004), a notion corroborated by models indicating improved outcomes with the absence of CD4 and CD8 cells (Yilmaz et al., 2006). Fever, another by-stander or prevalent potential contributor (Schwarz et al., 2000) to hematoma expansion and poorer outcomes, exacerbates brain metabolism and accelerates excitotoxicity, culminating in neuronal death (Commichau et al., 2003; Mayer and Rincon, 2005).

Therapeutic hypothermia has emerged as a potential intervention to mitigate secondary brain injury after ICH. Preclinical studies using systemic and local cooling techniques have shown promising results, including reductions in cerebral edema, blood-brain barrier disruption, and improved behavioral outcomes (Melmed and Lyden, 2017; Baker et al., 2022a). However, clinical trials exploring the efficacy of hypothermia have yet to yield conclusive evidence (Baker et al., 2022a).

The distinction between local and systemic hypothermia is a pivotal consideration in designing therapeutic trials and future interventions. While systemic hypothermia has been a predominant approach in both preclinical and clinical studies of ICH (Melmed and Lyden, 2017; Baker et al., 2022a), it has been associated with an elevated risk of adverse events (Lord et al., 2014). A substantial portion of preclinical studies have focused on localized hypothermia, which mitigates these drawbacks and may significantly contribute to the observed benefits in preclinical settings compared to clinical studies. Exploring diverse localized cooling techniques has yielded promising results in reducing brain temperature (Poli et al., 2013, 2014). Furthermore, a small clinical study demonstrated improved functional outcomes with the implementation of localized hypothermia (Su et al., 2015), underscoring the importance of addressing and obviating the adverse effects associated with systemic cooling.

Acknowledging the lack of standardized cooling techniques for ICH, international experts (Baker et al., 2022b) have proposed a four-step protocol combining systemic normothermia and localized hypothermia with minimally invasive surgical evacuation (MIS). The approach involves pre-operative systemic cooling (36.5°C), intra-operative focal cooling (33.5°C), post-operative focal cooling (33.5°C) combined with systemic surface cooling (36.5°C), and a gradual rewarming process (rate of 0.5°C every 6 h for 48 h). This strategy aims to utilize MIS to enable targeted hypothermia, which can address primary and secondary brain injury by reducing mass effect, eliminating clots, and curbing the inflammatory response while mitigating risks associated with systemic hypothermia. Despite being grounded in current clinical understanding, the effectiveness of this approach still needs to be verified through randomized controlled trials. The authors stress the need for well-designed clinical trials to determine the best cooling protocols for ICH when using MIS. They outline the criteria for patient selection and stress the importance of conducting feasibility, pilot, and pivotal trials. Similar yet modified cooling protocols could be adopted in future problems not involving surgical evacuation, emphasizing the importance of identifying the most effective device for precise brain cooling. There are no consensus statements for stand-alone cooling protocols in ICH.

Commichau et al. (2003) found improved functional outcomes by combining cooling and hematoma evacuation, although the follow-up was limited to 7 days post-surgical intervention. The TTM-ICH trial (Rincon et al., 2014) and the CINCH trial (Kollmar et al., 2012) will provide additional insights into the effects of therapeutic hypothermia on intracerebral hemorrhage (ICH) outcomes.

This review article aimed to offer an up-to-date synopsis of the existing literature concerning the application of hypothermia in patients with intracerebral hemorrhage (ICH). However, it is essential to acknowledge the inherent limitations of this review. First, this review cannot establish causative relationships due to its nature, as it relies on existing studies. Moreover, the presence of selection bias and the differences in the study populations and methodologies across the individual studies contribute to inherent heterogeneity, which can hinder the generalizability of the findings. Despite these limitations, this work provides a comprehensive overview of the mechanisms of injury and potential therapeutic avenues in the context of intracerebral hemorrhage.

While the potential benefits of hypothermia as a therapeutic approach for ICH are promising, challenges remain in translating preclinical success into clinical efficacy. Heterogeneity in study designs, cooling methods, temperature targets, initiation timing, and cooling duration (“dose”) contribute to the lack of consensus in the existing literature (Baker et al., 2022a,b). Future research efforts should focus on refining hypothermia protocols, incorporating localized cooling strategies, and conducting well-designed, randomized clinical trials to establish the safety and effectiveness of hypothermia as a treatment for ICH and its related complications.

In conclusion, ICH still poses a significant clinical challenge due to its high morbidity and mortality. The intricate interplay of primary and secondary brain injury drives the devastating outcomes associated with ICH. Therapeutic hypothermia offers a potential avenue for intervention by targeting the secondary injury mechanisms. While preclinical studies have shown promising results, clinical trials have yet to provide definitive evidence of hypothermia’s efficacy. The ongoing pursuit of optimized cooling protocols, localized cooling strategies, and rigorous clinical trials is crucial to unlocking the potential of hypothermia as a therapeutic tool for managing ICH and improving patient outcomes.

## Author contributions

AC: Writing−original draft, Writing−review and editing. FR: Conceptualization, Supervision, Writing−review and editing.
